# Avoiding scar tissue formation of peripheral nerves with the help of an acellular collagen matrix

**DOI:** 10.1371/journal.pone.0289677

**Published:** 2023-08-04

**Authors:** Martin Aman, Maximilian Mayrhofer-Schmid, Daniel Schwarz, Martin Bendszus, Simeon C. Daeschler, Tess Klemm, Ulrich Kneser, Leila Harhaus, Arne H. Boecker

**Affiliations:** 1 Department of Hand-, Plastic and Reconstructive Surgery, Burn Center, BG Trauma Center Ludwigshafen, Department of Hand- and Plastic Surgery, University of Heidelberg, Heidelberg, Germany; 2 Department of Neuroradiology, Heidelberg University Hospital, Heidelberg, Germany; University College London Institute of Child Health, UNITED KINGDOM

## Abstract

**Introduction:**

Extensive scar tissue formation after peripheral nerve injury or surgery is a common problem. To avoid perineural scarring, implanting a mechanical barrier protecting the nerve from inflammation processes in the perineural environment has shown promising results for functional recovery. This study investigates the potential of an acellular collagen-elastin matrix wrapped around a peripheral nerve after induction of scar tissue formation.

**Materials and methods:**

In the present study, 30 Lewis rats were separated into three groups and sciatic nerve scarring was induced with 2.5% glutaraldehyde (GA-CM) or 2.5% glutaraldehyde with a supplemental FDA-approved acellular collagen-elastin matrix application (GA+CM). Additionally, a sham group was included for control. Nerve regeneration was assessed by functional analysis using the Visual Statisc Sciatic Index (SSI) and MR neurography during the 12-week regeneration period. Histological and histomorphometry analysis were performed to evaluate the degree of postoperative scar tissue formation.

**Results:**

Histological analysis showed an extensive scar tissue formation for GA-CM. Connective tissue ratio was significantly (p < 0.009) reduced for GA+CM (1.347 ± 0.017) compared to GA-CM (1.518 ± 0.057). Similarly, compared to GA+CM, MR-Neurography revealed extensive scar tissue formation for GA-CM with a direct connection between nerve and paraneural environment. Distal to the injury site, quantitative analysis presented significantly higher axon density (p = 0.0145), thicker axon diameter (p = 0.0002) and thicker myelinated fiber thickness (p = 0.0008) for GA+CM compared to GA-CM. Evaluation of functional recovery revealed a significantly faster regeneration for GA+CM.

**Conclusion:**

The supplemental application of an acellular collagen-elastin matrix showed beneficial effects in histological, radiological, and functional analysis. Therefore, applying a collagen-elastin matrix around the nerve after peripheral nerve injury or surgery may have beneficial effects on preventing scar tissue formation in the long run. This represents a feasible approach to avoid scar tissue formation in peripheral nerve surgery.

## Introduction

Perineural scar tissue formation following nerve surgery is a relevant problem frequently resulting in severe functional impairment and pain. Especially extensive reconstructive attempts can be hampered by the development of scar tissue. In the literature, external scarring is frequently referred to as a perineural or paraneural scar, relating to fibrosis of the nerve’s surrounding tissue. In the following, the formation of perineural scar tissue represents the external scarring of the peripheral nerve. Extensive external scar tissue formation lead to nerve’s adhesion to its surrounding tissue. As a result functional results are often limited, particularly in the vincinity of joints. Therefore, avoiding excessive scarring is an essential aim in peripheral nerve surgery.

Numerous strategies to prevent severe scar tissue formation have been examined in the literature. Ranging from supplementary pharmacological applications to surgical refinements or supplementary application of spacer materials to protect the site of the nerve lesion [1]. The latter has shown promising results in modifying the inflammation response after nerve injury [2]. Therefore, the ideal spacer to prevent extensive scar tissue formation should lead to a decreasing inflammatory reaction during biodegradation, providing acceptable porosity, and reducing nerve adhesion. Specifically, protection strategies can be divided into two approaches, using either autologous or synthetic material. Autologous materials, such as veins or a local adipose cutaneous fat pad, are placed around the nerve to avoid nerve adhesion by building a biological barrier between the nerve and the perineural environment [3]. As biological substitutes, collagen membranes [4] and hyaluronic acid carboxymethylcellulose films [5] are designed to build up a barrier after implantation and reduce scar tissue formation. Although there is a variety of materials described in preclinical studies to decrease scar tissue formation, their transition clinical application is limited. Most described clinical uses for these materials are in the treatment of chronic and recurrent compression neuropathies and not in the primary use for the avoidance of scar tissue formation in a preventive setting [6]. Keeping in mind the importance of avoiding postoperative scar tissue formation, a clinical gold standard is yet to be found.

Collagen, in particular, is a well-established material in plastic surgery due to its biocompatibility. In contrast to other materials, such as polyglycolic acid, collagen exhibit toxic-free biodegradation without inducing further scar tissue formation [7]. Based on encouraging results in other fields of reconstruction (e.g. nerve conduits) and the strong clinical need to avoid perineural scar tissue formation, we see a useful adhesion protection potential of this material. Therefore, we examined a collagen-elastin matrix as a spacer material to prevent significant external scar tissue formation in the rat sciatic nerve model.

## Materials and methods

### Animal model and surgical technique to induce scar tissue formation

**Fig 1:**
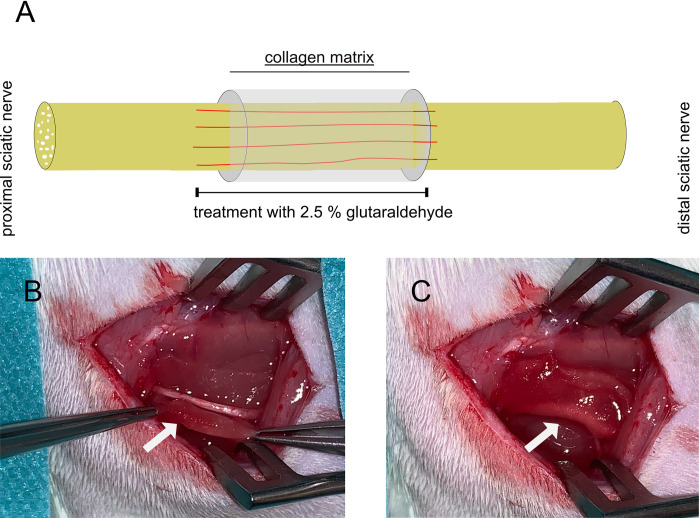
Intraoperative placement of the collagen-elastin matrix. The placement of the collagen-elastin matrix is depicted schematically. Due to the matrix’s adhesion to the sciatic nerve, no additional fixation is required. Red lines indicate the area of the GA application (A). Intraoperative placement of the acellular collagen-elastin matrix behind (B) and then wrapped around the injured sciatic nerve (C); white arrows indicate the collagen-elastin matrix.

All animals received humane care according to FELASA principles, and local ethics board (Landesuntersuchungsamt Rheinland-Pfalz, Koblenz, Germany) approval was obtained under the number G 20-7-004. For this study, the sciatic nerve model was used. A total of 30 female Lewis rats were randomized into three groups with ten animals each. Group 1 underwent perineural scar induction with subsequent implantation of a collagen-elastin matrix around the nerve injury site (GA+CM, treatment group), Group 2 underwent perineural scar induction only (GA-CM, negative control), and Group 3 had a sham procedure (positive control). To induce scar tissue formation, 10 μl of 2.5% glutaraldehyde was applied (GA) using a pipet over an area of 10mm onto the sciatic nerve (GA-CM) according to the protocol of Lemke et al. [8]. For animals treated with the collagen-elastin matrix (GA+CM), the acellular collagen-elastin matrix was placed around the nerve after glutaraldehyde application (see Fig 1). Due to the adhesion of the collagen-elastin matrix to the nerve, no further fixation was needed. For the sham group, the sciatic nerve was mobilized from its tissue bed, as in the other groups, without further intervention.

Experiments were performed in a blinded fashion as the surgeon performing the experiments received a randomly picked rat after delivery by an assistant who was blind which animal received which treatment. During further analysis during the experiments, one person blinded to the treatment for each animal performed functional as well as histological analysis. Functional analysis was performed weekly, MR neurography was performed at 6 and 12 weeks postoperatively and the re-exploration of the sciatic nerve for histological specimen extraction was done 12 weeks postoperatively.

### Acellular collagen-elastin matrix

The acellular collagen-elastin matrix MatriDerm^®^ (MedSkin Solutions Dr. Suwelack AG, Germany), is a three-dimensional matrix composed of native structurally intact collagen-elastin fibrils and elastin inteded to support tissue regeneration. The collagen is obtained from bovine dermis and contains dermal collagen type I, III and V. It is manufactured using a proprietary method that results in a more natural, native collagen-elastin matrix with no chemical cross-linking.

### Functional analysis with Visual SSI

Recovery of motor function was measured by the Visual Static Sciatic Index (SSI) preoperatively and weekly throughout the postoperative period until the end of the experiment [9]. Animals were placed in a plexiglass container, and the toe spread between the first and the fifth digit, as well as the intermediate toe spread (ITS) between the second and the fourth digit were assessed via a camera placed beneath. The toe spread and the intermediate toe spread factors were used to calculate the static sciatic index.

### MR neurography

MR neurography was conducted as previously reported 1. Rats were anesthetized with 3% isoflurane and maintained with 1–2% isoflurane [10]. The animals were placed supine on a heating pad to maintain their body temperature. External respiration was measured using a breathing surface pad controlled by a customized LabView application (National Instruments Corporation, Austin, TX, USA).

MR imaging was conducted on a 9.4 Tesla horizontal bore small animal NMR scanner (BioSpec 94/20 USR, Bruker BioSpin GmbH, Ettlingen, Germany) with a four-channel phased-array surface receiver coil. The MR protocol included the following sequence settings:

1. High-resolution T2-w Rapid Acquisition with Refocused Echoes (RARE) sequence with flip-back technique: 2D sequence, echo time (TE): 40 ms, repetition time (TR): 2500 ms, rare factor 4, spectral fat saturation, 55 μm x 55 μm in plane resolution, acquisition matrix: 512 x 182, number of slices: 17, slice thickness: 1 mm, number of averages: 2, acquisition time: 4 min 35 s.
2. High-resolution T1-w Fast Low-Angle Shot (FLASH) sequence before and after intravenous administration of the contrast agent gadoteric acid (Dotarem, Guerbet, 0.2 mmol/kg): 2D sequence, TE: 4.7 ms, TR: 368 ms, 55 μm x 57 μm in plane resolution, acquisition matrix: 277 x 210, number of slices: 17, slice thickness: 1 mm, number of averages: 4, acquisition time: 10 min 17 s.

### Histological assessment

To evaluate the development of scar tissue 12 weeks postoperatively, 5 μm thick, paraffine-embedded nerve cross sections were stained with Masson’s Trichrome and Picro-Sirius-Red. ImageJ (RSB, imagej.nih.gov/ij) was used to quantify the connective tissue area (epineurium, perineurium, and scar tissue) and the cross sectional fascicular area using 20x enlarged digitalized pictures. We utilized the ratio of total fascicular area to total connective tissue area to quantify scar tissue area.

### Histomorphometric analysis

For histomorphometric analyses, nerve tissue directly distal to the injury site and the contralateral, uninjured distal sciatic nerve were stained using osmium tetroxide and embedded in paraffin. The embedded samples were trimmed to 2μm thin cross sections and the paraffin removed from the mounts holding the samples. Samples were analyzed using a 40x objective of a Zeiss Axio Imager 2 (Carl Zeiss MicroImaging GmbH, Goettingen, Germany) and ImageJ.

First, the area of the whole nerve cross section was determined. Then one representative third of the nerve was chosen for manual analysis. After measuring the area of this third, all axons within were counted to determine axon density. Afterwards, all fibers in this area were measured for their axonal thickness and the myelin sheath thickness. Myelinated fiber thickness and g-ratio were calculated.

### Muscle weight analysis

The gastrocnemius and tibialis anterior muscles, both target muscles of the injured sciatic nerve, were harvested from the operated and the contralateral side of all animals. After standardized resection of surrounding connective tissue, wet muscle weight was analyzed using an Adventurer Pro analytical scale (Ohaus Europe GmbH, Nänikon, Switzerland). To avoid animal weight-related distortions, a weight ratio of the operated side to the contralateral side was used to determine differences in wet muscle weight between groups.

### Statistical analysis

All statistical analysis were performed using Prism 9 (GraphPad Software, San Diego, California USA). The Shapiro-Wilk test was used to assess all data for normal distribution. Statistical significance was assessed using a one-way ANOVA with a subsequent Tukey’s multiple comparisons test to compute P-values for pairwise comparisons. P- values of <0.05 were considered as statistically significant.

For the Visual SSI, a repeated measurements ANOVA was used to compare measurements at different timepoints within the same group.

## Results

### Functional analysis with Visual SSI

**Fig 2:**
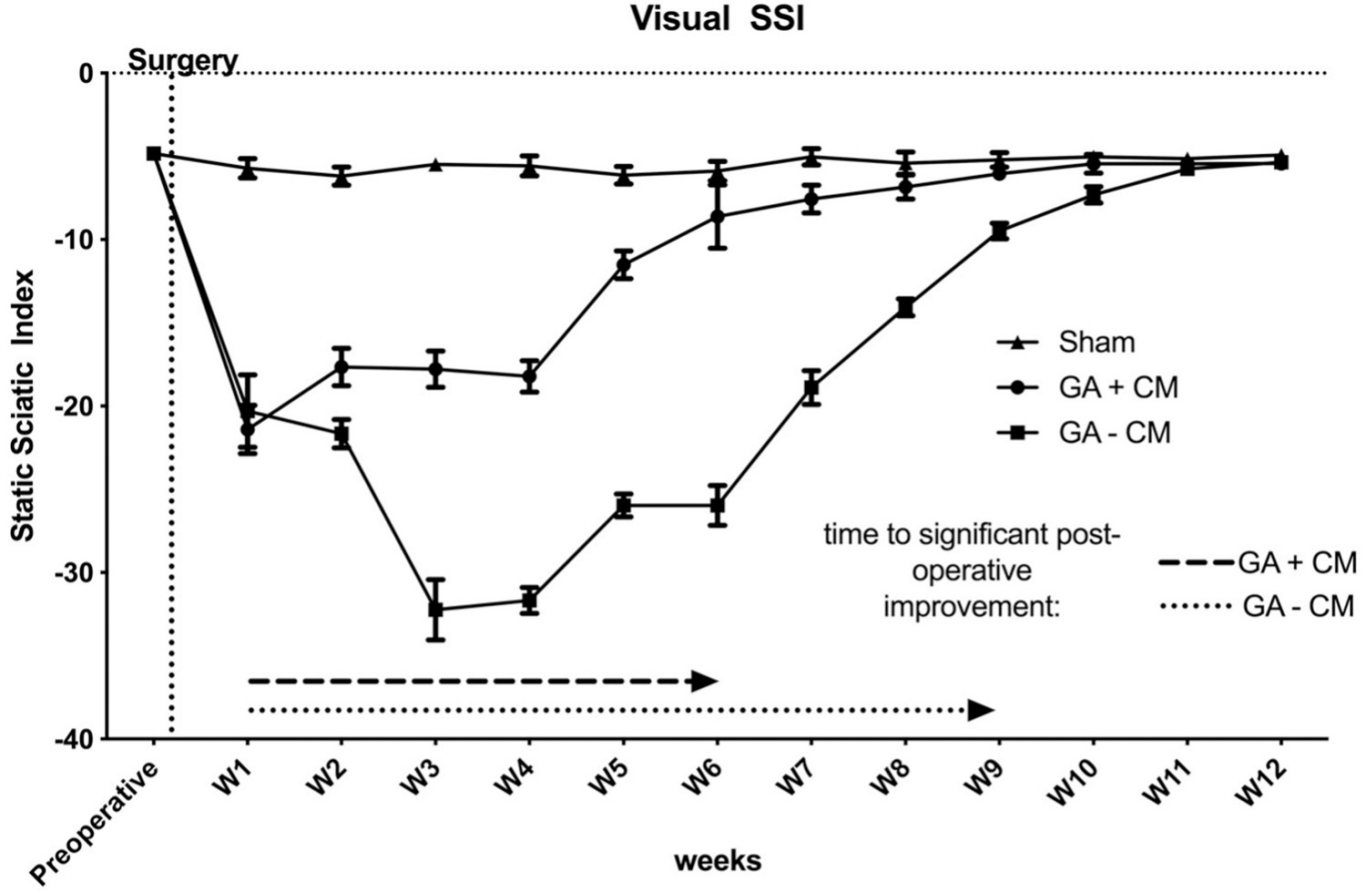
Visual static sciatic index during 12 weeks regeneration time. Functional regeneration improved over the regeneration period significantly after six weeks for GA+CM and three weeks earlier than GA-CM. GA+CM presented significantly better functional recovery between weeks two to ten than GA-CM.

The SSI dropped significantly (P < 0.01) directly postoperative for GA-CM and GA+CM groups (GA-CM: -21.399 ± 1.458; GA+CM: -20.305 ± 2.176) (see Fig 2). The sham group showed no functional impairment after the operation (Sham: -5.723 ± 0.594). GA-CM showed a significant (p = 0.012) further decrease in function with -21.650 ± 0.868 two weeks postoperatively compared to GA+CM (SSI = -17.658 ± 1.131). Results of GA+CM presented significantly better results than GA-CM until the 10th week during the regeneration period (GA+CM: -5.435 ± 0.569; GA: -7.306 ± 0.489). Compared to the postoperative first-week results (-20.305 ± 2.176), functional regeneration was obtained after nine weeks for GA-CM (-9.492 ± 0.478). In the final analysis after 12 weeks, no significant differences could be detected comparing Sham and GA-CM (P = 0.505) as well as between Sham and GA+CM (P = 0.151). GA+CM revealed first regeneration after 6 weeks compared to Sham (GA+CM: -8,606 ± 1,909; Sham: -5,878 ± 0,5734; P = 0.188) as well as postoperative 1st week results (-8.606 ± 1.909).

### Visualization of perineural scarring with MRI

**Fig 3:**
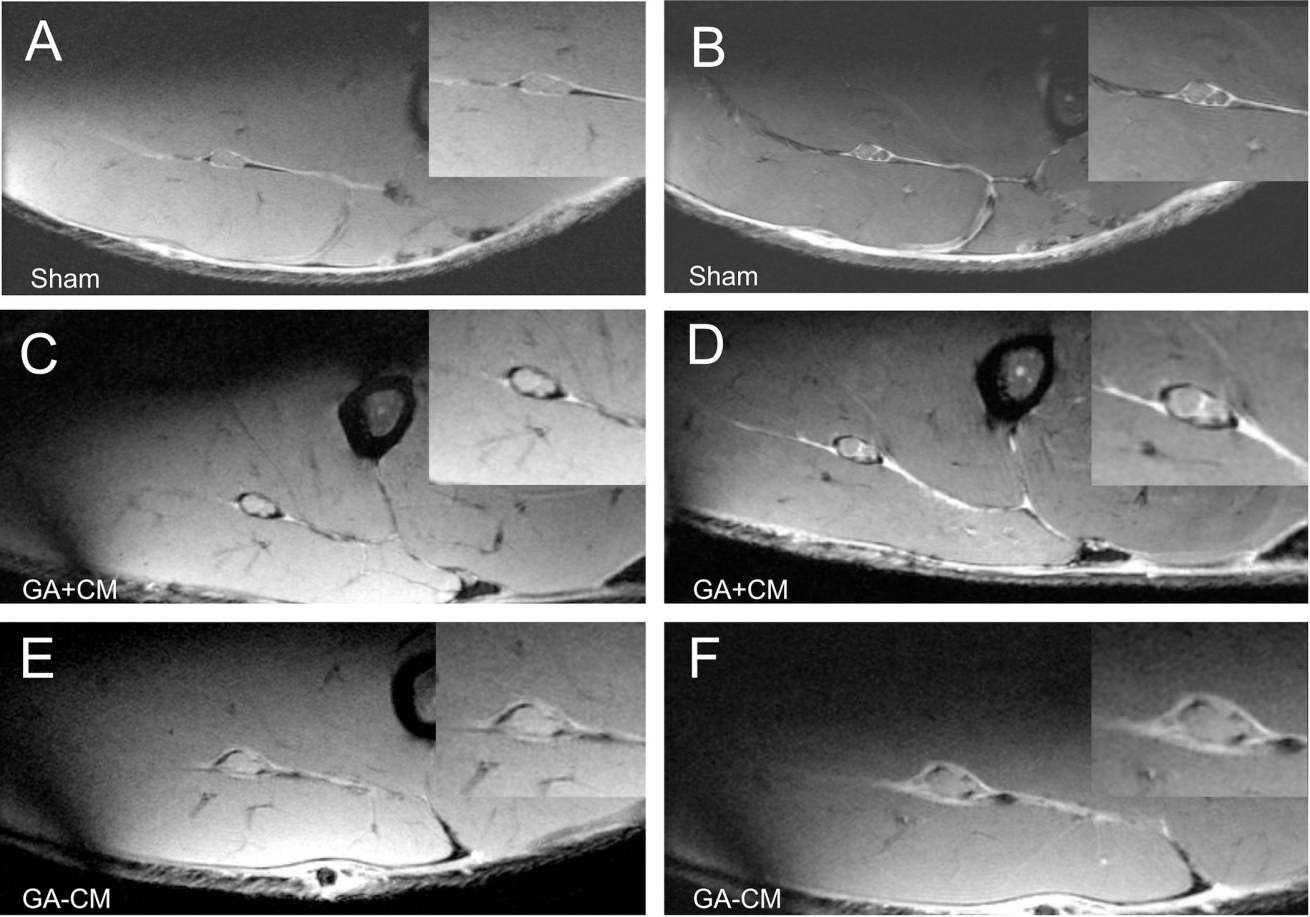
Analysis of the MR-Neurography 6 weeks postoperatively. The supplemental application of CM affects the formation of scar tissue. Unlike GA+CM (Images C-D), scar formation directly surrounded the sciatic nerve in rats for GA-CM (Images E-F). Additionally, the fascicular structure is less detectable for animals without additional CM compared to GA+CM (see magnification F). Images were acquired using the following settings: High-resolution T2-w Rapid Acquisition with Refocused Echoes (RARE) sequence with flip-back technique (Images A, C, E) and high-resolution T1-w Fast Low-Angle Shot (FLASH) sequence after intravenous administration of the contrast agent gadoteric acid (Images B, D, F).

After six weeks, no scarring was noted in the Sham group (see Fig 3A and 3B).

**Fig 4:**
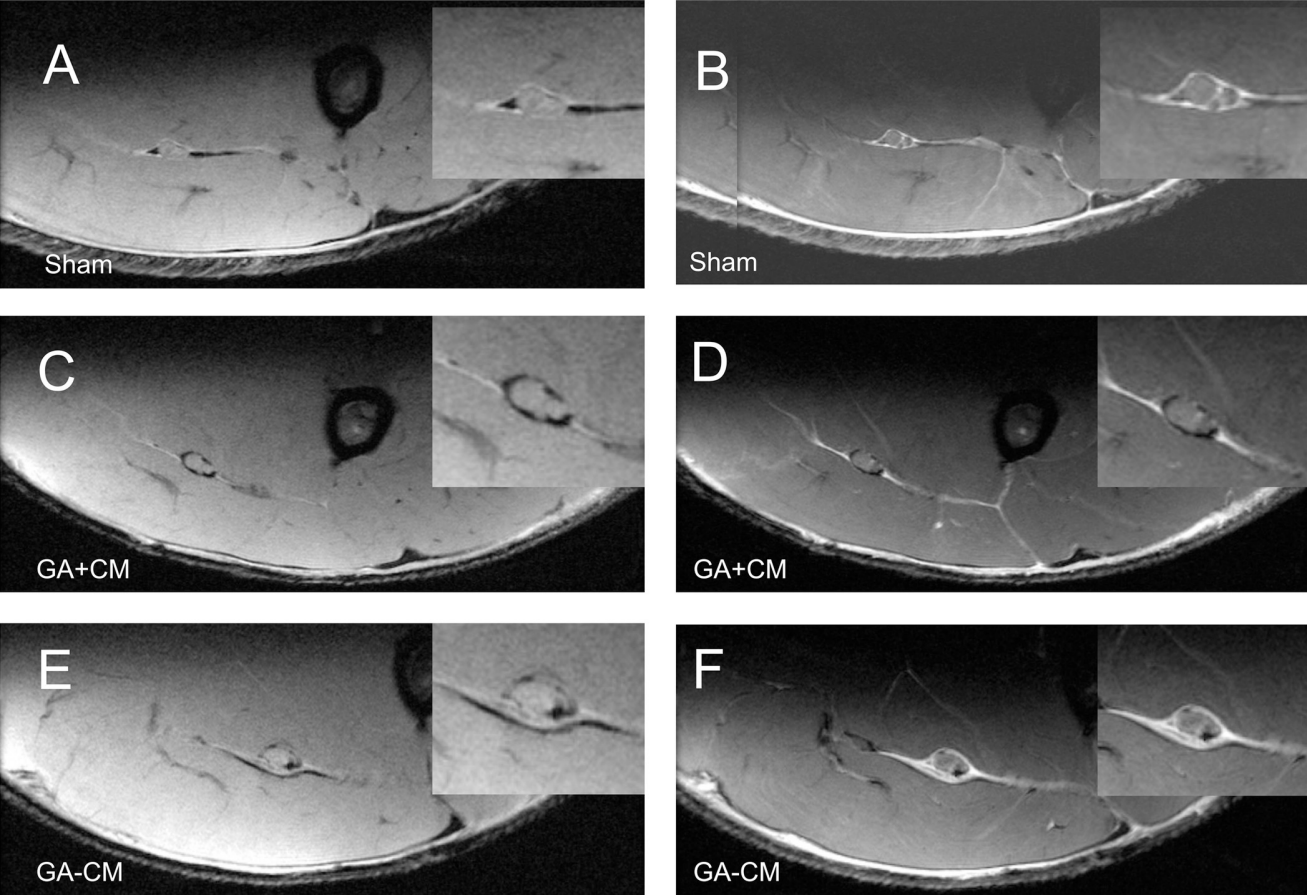
Analysis of the MR-Neurography 12 weeks postoperatively. After 12 weeks, scar tissue formation increased considerably in both GA+CM and GA-CM animals. Compared to GA-CM, GA+CM remnants of the collagen-elastin matrix may inhibit direct attachment of the sciatic nerve to the soft tissue surroundings (Images C-D). Images were acquired using the following settings: High-resolution T2-w Rapid Acquisition with Refocused Echoes (RARE) sequence with flip-back technique (Images A, C, E) and high-resolution T1-w Fast Low-Angle Shot (FLASH) sequence after intravenous administration of the contrast agent gadoteric acid (Images B, D, F).

For GA+CM, the extent of perineural scar tissue formation was more extensive as evidenced by the more intense interstitial enhancement on post-contrast images. Interestingly, the CM material could still be detected seemingly preventing excessive perineural tissue growth (see Fig 4C and 4D). However, in the GA-CM group substantial scar tissue formation could be observed six weeks(see Fig 4E and 4F). After 12 weeks, scar formation showed some further increase during the regeneration period in GA+CM and GA-CM (see Fig 3). Remnants of CM could still be observed.

### Intraoperative findings

**Fig 5:**
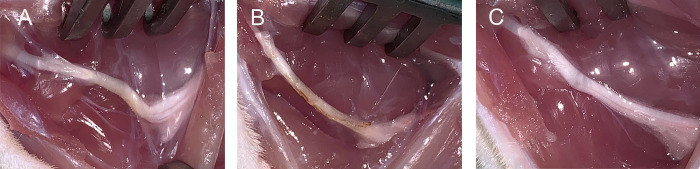
Intraoperative findings 12 weeks postoperatively of GA-CM, GA+CM and Sham group. A: GA-CM, B: GA+CM, C: Sham. Exposed sciatic nerve over the distance of 2 cm after a regeneration period of 12 weeks, showing external scar tissue formation with signs of adhesion in the surrounding tissue (A). Small residua of the collagen-elastin matrix were macroscopically visible for GA+CM. No signs of severe scar tissue formation or extended adhesion of the sciatic nerve were found (B). Sham group showed macroscopically no scar tissue formation or nerve adhesion after 12 weeks postoperatively (C).

After exploring the rat’s sciatic nerve after 12 weeks, GA revealed external scar tissue development and nerve adhesion to the surrounding tissue. Macroscopically, GA+CM showed no signs of serve scar tissue formation. No external scar development nor nerve adhesion were seen in the sham group (see Fig 5A–5C).

### Histomorphometry and perineural scarring

**Fig 6:**
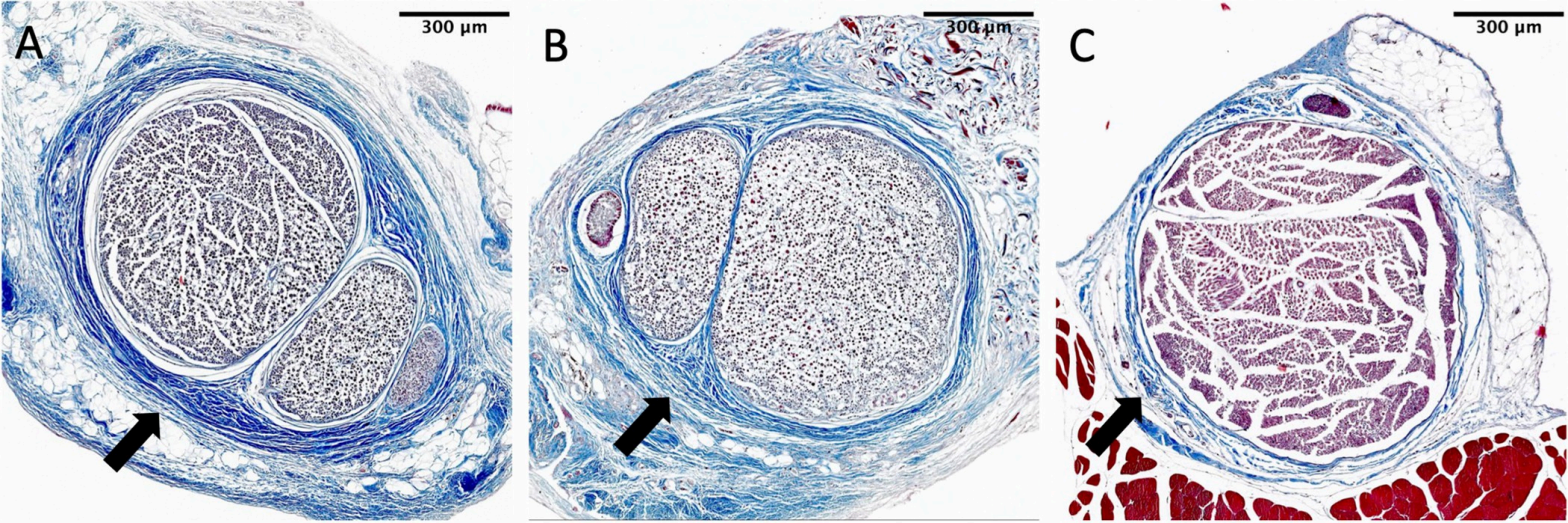
Histological findings of scar tissue formation after treatment with the acellular collagen-elastin matrix. A: GA-CM, B: GA+CM, C: Sham. Visibly more severe perineural scarring is observed with GA-CM (A) compared to GA+CM (B) and Sham (C; see black arrow).

The histological staining showed increased perineural scar tissue formation for GA-CM compared to GA+CM (see Fig 6A). Slightly reduced thickness of the scar tissue was observed after applying an acellular collagen-elastin matrix wrapped around the sciatic nerve (see Fig 6B). There is nearly no scar tissue formation for animals treated with sham surgery (see Fig 6C).

In qualitative analysis, microscopical findings presented a thicker perineural scar tissue formation for GA-CM compared to GA+CM and Sham.

**Fig 7:**
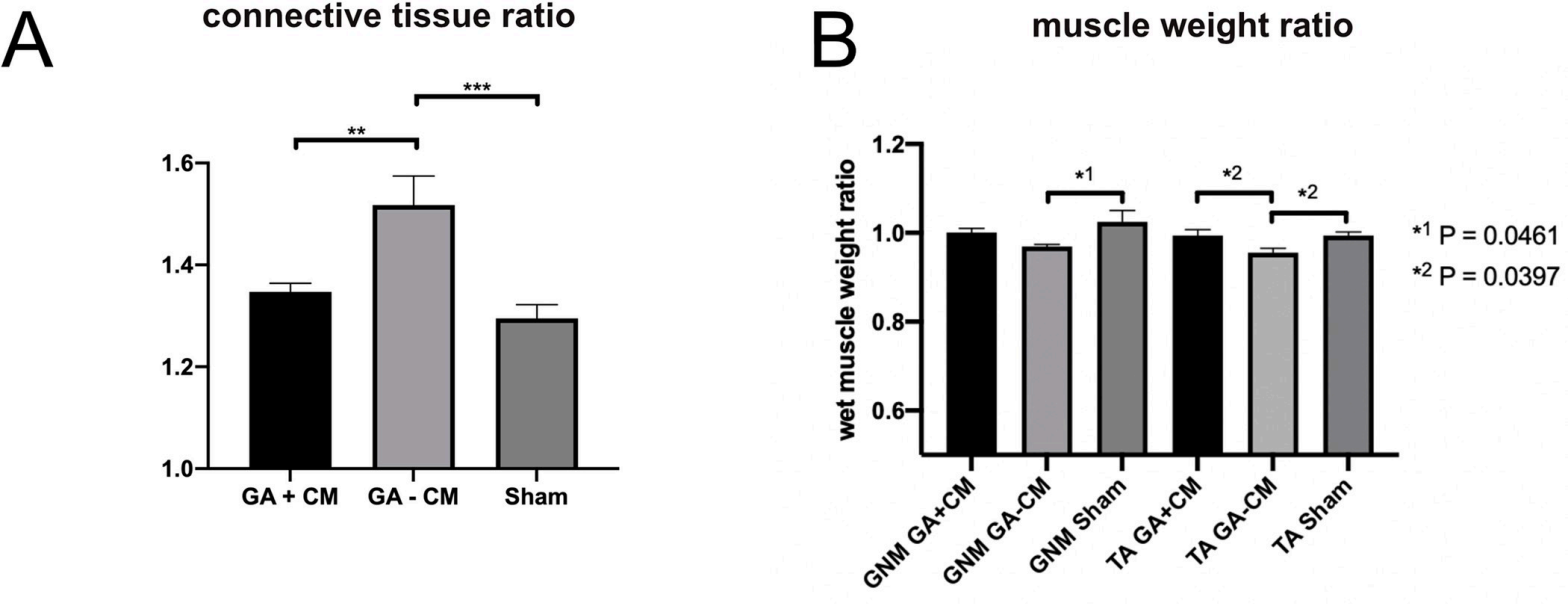
Analysis of perineural scar formation and muscle weight ratio analysis 12 weeks postoperatively. In comparison to GA-CM, the use of supplemental CM considerably reduced perineural scarring. Interestingly, the ratio of scar tissue of GA+CM presented outcomes similar to sham without showing significant difference (A). Muscle weight ratio showed a slightly decreased muscle weight ratio without supplemental CM application in the tibialis anterior muscle, but not in the gastrocnemius muscle.(B). GNM: gastrocnemius muscle, TA: anterior tibialis muscle.

In quantitative study, histological data revealed severe perineural scarring in glutaraldehyde-treated rats, with connective tissue ratio values of 1.518 ± 0.057. After 12 weeks, GA+CM showed a significant decrease in perineural scarring (1.347 ± 0.017) compared to GA-CM (P = 0.009). As a control group, the sham group did not develop relevant scar tissue, with values of 1.295 ± 0.027 (see Fig 7A).

Comparing the postoperatively assessed wet muscle weights using a ratio between the operated and the non operated side, we found GA-CM to have a slightly decreased muscle weight ratio for the gastrocnemius muscle compared to Sham (P = 0.0461). In the tibialis anterior muscle ratio, GA-CM showed a decreased muscle weight when compared to both, GA+CM and sham (for both: P = 0.0397) (see Fig 7B).

### Histomorphometry analysis

Histomorphometric analysis of cross sections distal to the site of injury demonstrated significantly higher axon density (p = 0.0145) in GA+CM with 11882 ± 541 fibers/mm^2^ compared to GA-CM with 9955 ± 471 fibers/mm^2^ and comparable outcomes to Sham (11659 ± 419 fibers/mm^2^) (see Fig 9A). Axons in the GA+CM group were significantly lager (p = 0.0002) with a diameter of 4.275 ± 0.1 μm compared to 3.64 ± 0.1 μm for GA-CM. The sham group had comparable results to the GA+CM group with 4.25 ± 0.13 (see Fig 9B). The difference in myelin sheath thickness between GA+CM (1.491 ± 0.08) and GA-CM (1.35 ± 0.05) was not statistically significant (p = 0.187). No differences between Sham (1.48 0.04) and GA+CM were also evident (p = 0.995) (see Fig 9C). In contrast, the myelinated fiber thickness of GA+CM (7.26 ± 0.21) revealed thicker nerve fibers with a significant difference (p = 0.0008) to GA-CM (6.34 ± 0.08). Sham group has findings comparable to GA+CM and a much larger myelinated axon diameter than GA-CM (7.22 ± 0.20) (see Fig 10A). The calculated g-Ratio parameter revealed no significant changes across groups (GA+CM 0.59 ± 0.01, GA-CM 0.57 ± 0.01, and sham 0.58 ± 0.001) (p = 0.460 and p = 0.967) (see Fig 10B).

## Discussion

After peripheral nerve injury or nerve surgery, scar tissue development can lead to nerve adhesion and potentially nerve compression. Both can significantly affect nerve regeneration and function due to a reduced perfusion following axonal regeneration. Furthermore, besides functional impairment, nerve compression can lead to neuropathic pain, which negatively affects the patient’s quality of life.

Therefore, as patients undergoing nerve surgery are generally at risk for an impaired and sometimes unpredictable outcome, refinement of surgery is of utmost importance.

Numerous strategies have been tested to prevent recurrent nerve compression by implanting autologous or synthetic spacer materials, including veins or fibrin glue, to protect the nerve lesion site [3, 11, 12]. Besides synthetic materials like polyglycolic acid or poly- DL-lactide-caprolactone, collagen-based materials are well known in nerve-conduit research and clinical practice [13]. Collagen in this field has the potential for a tailored architecture to mimic the peripheral nerve while degrading into nontoxic byproducts without causing additional scar tissue [14, 15]. This is consistent with our findings, which indicate that the collagen-elastin matrix was nearly completely resorbed twelve-weeks post surgery as shown in our histomorphometrical and MRI analysis (see Figs 3–5). Due to the promising results for collagen in the field of peripheral nerve surgery [7] in general, and as spacer material [4, 16] in particular, we were encouraged to investigate the potential of the acellular collagen-elastin matrix Matriderm^®^ (CM) to prevent scar tissue formation after nerve trauma.

Interestingly, CM appears to reduce scarring, despite its rapid degradation. Histological findings showed that there is significantly less perineural scarring for GA+CM, and the collagen-elastin matrix seems to preserve a small space between the nerve and its surrounding tissue as seen in the MR-Neurography compared to GA-CM (see Figs 3–7). This is in line with the preliminary findings of Kim et al., which demonstrated a significant decrease in connective tissue formation in animals with a collagen conduit wrapped around the nerve’s transection site. However, in this study there were no significant differences in axon density, myelin sheath thickness, and myelinated fiber diameter [14].

**Fig 8:**
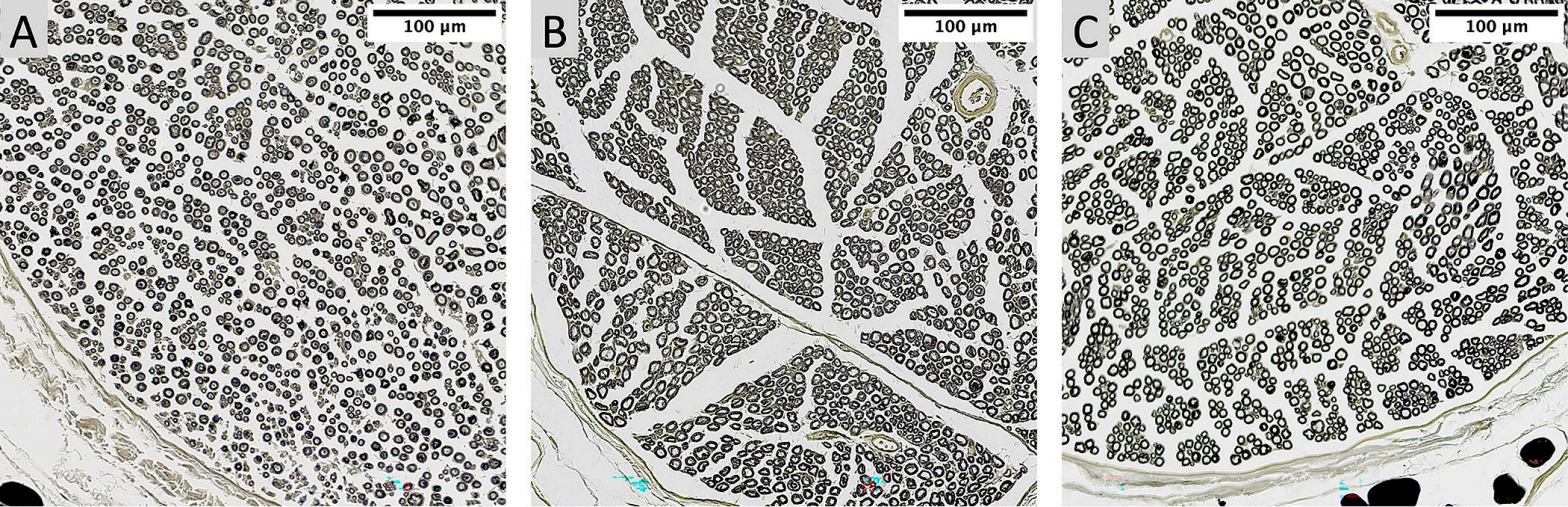
Nerve cross sections used for histomorphometric analysis. Three exemplary sections of images used for histomorphometric analysis stained with osmium tetroxide are displayed. In quantitative analysis, GA-CM (A) demonstrated thinner axons, a lower myelinated fiber thickness and a lower axon density than GA+CM (B) and Sham (C).

**Fig 9:**
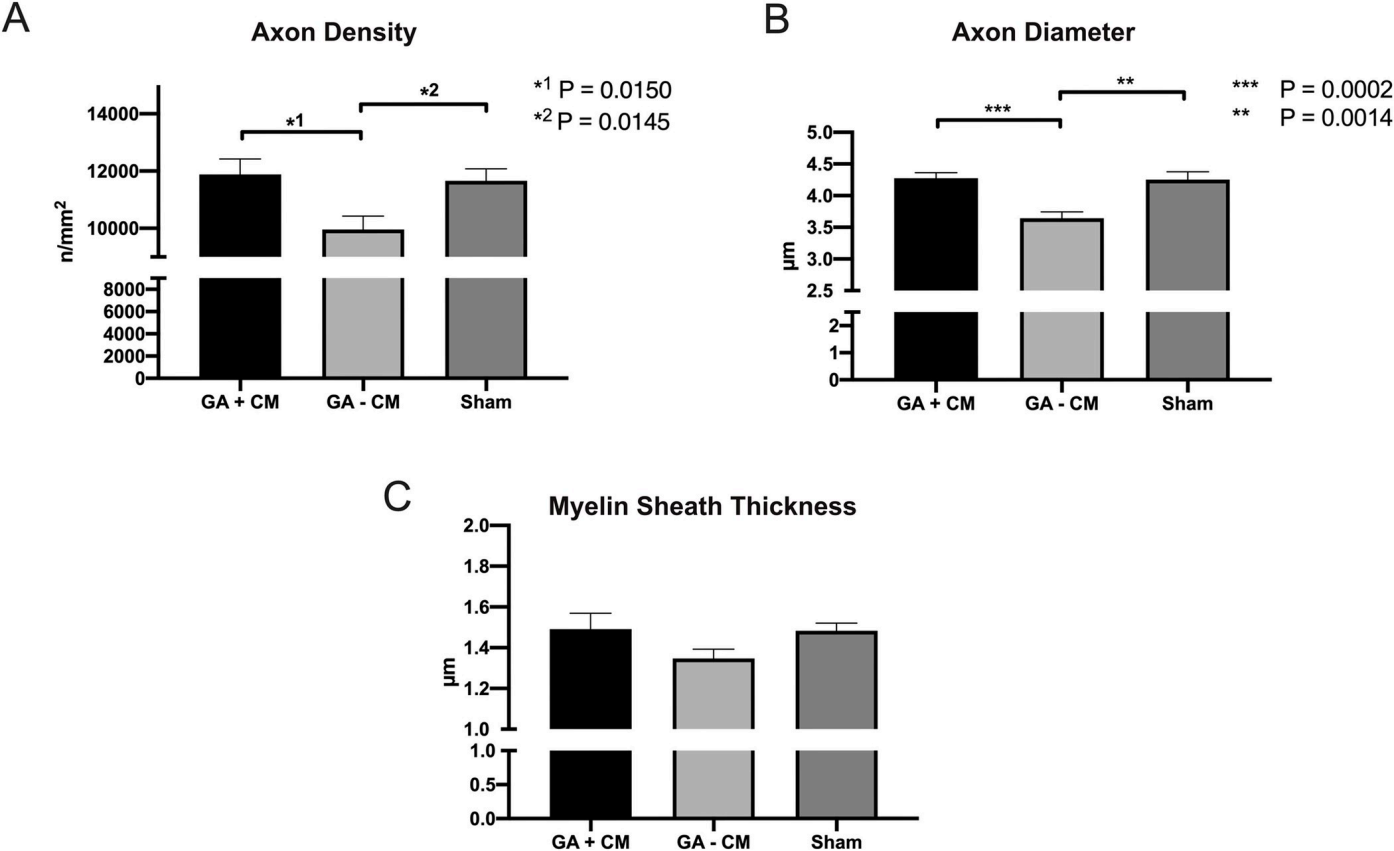
Histomorphometry analysis of the rat’s sciatic nerve. Compared to GA-CM, the analysis for GA+CM and Sham showed a significant increase in axon density (A). Axon diameter also revealed a significantly thicker diameter for GA+CM and Sham than GA-CM (B). All groups had no significant differences in myelin sheath thickness (C).

**Fig 10:**
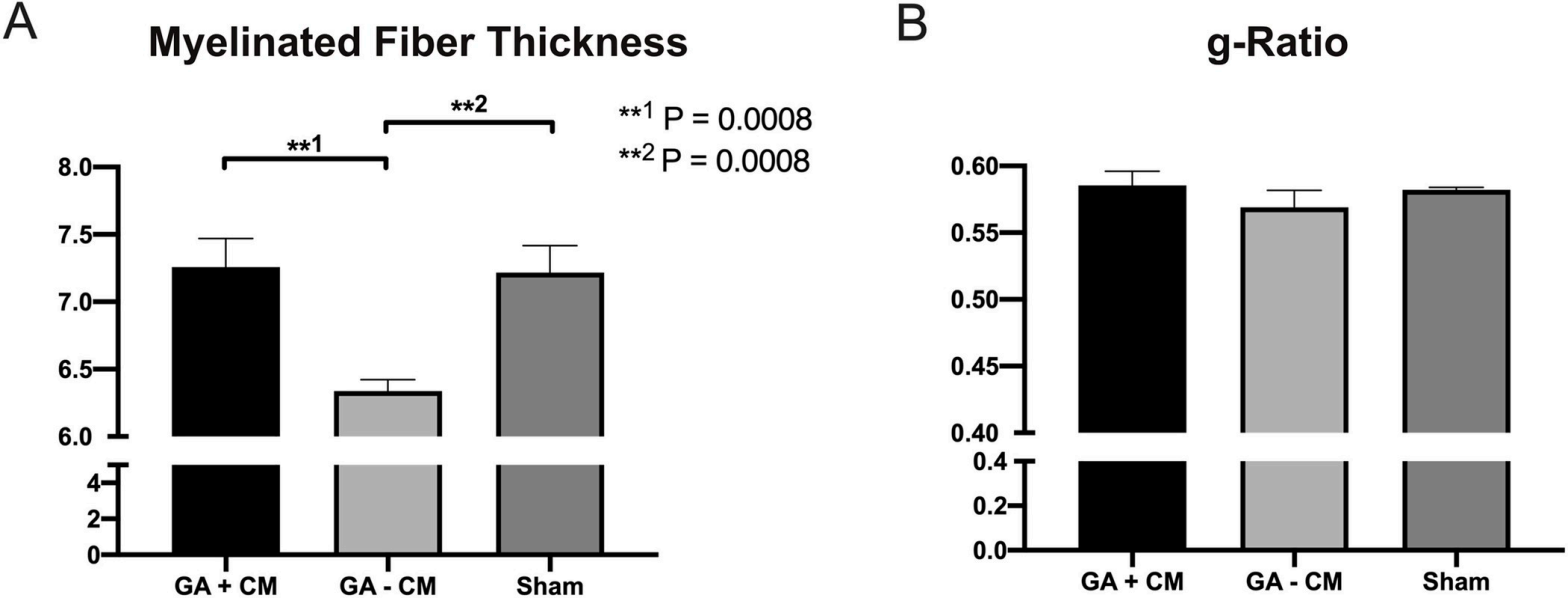
Histomorphometry analysis of the rat’s sciatic nerve. Myelinated fiber thickness was signifacntly larger for GA+CM (A) compared to GA-CM and similar to Sham. In contrast, g-Ratio showed no significant differences between all groups (B).

Interestingly, as seen by the significantly higher fiber density and thicker axon diameter for GA+CM compared to GA-CM and even equivalent to Sham, the effects are not only restricted to the nerve’s connective tissue but also affect nerve fiber morphology (see Figs 8–10). Therefore, in contrast to the findings of Mathieu et al. and Kim et al., we observed a protective effect of CM application on regenerating nerve fibers [4, 14]. A possible explanation might be the different injury models used in these studies. Kim and Mathieu et al. transected the rat’s sciatic nerve to induce scar tissue formation. Compared to our study with the application of GA, the created nerve damage by nerve transection is far more severe and may obscure the effects of the additional application of collagen-elastin matrix in the histological evaluation.

Notably, the beneficial effects are not limited to histological changes but also translate to improved functional recovery. We observed that animals subjected to GA+CM recovered significantly faster with the first significant improvement after six weeks postoperatively, in contrast to GA-CM which showed first significant improvement after 9 weeks postoperatively (see Fig 2). Interestingly, it seems to be a direct correlation between the development of scar tissue formation, the histological and the estimated functional recovery. In accordance with Atkins et al., not only the development but also the quantity of scar tissue formation directly influence peripheral nerve regeneration [17]. However, after 12 weeks, the function fully recovered after GA application, independently of whether a supplementary acellular collagen-elastin matrix is used or not [18]. Simultaneously, however, some of the evaluated histomorphometric parameters in the GA-CM group had not yet returned to a level similar to sham. The decreased myelin fiber thickness and fiber density in the GA-CM group are not reflected in the functional assessment. This might either be explained by a different sensitivity to small changes between the assessment methods or by a faster functional regeneration with a lacking full recovery in the microscopical structure of the nerve. Similarly, Luis et al. evaluated the functional and histomorphometric recovery after a rat sciatic nerve crush injury and described a full recovery in the sciatic functional index after 7 weeks, with pathological changes in the histomorphometric evaluation still present at 12 weeks [19].

On this note, it is essential to underline that regeneration processes differ in rats and humans [16], resulting in a higher regenerative potential for different peripheral nerve injury models in rats. Therefore, the complete functional regeneration in both the GA-CM and the GA+CM group can most probably not be translated to human modalities.

Nevertheless, the significantly improved regeneration after CM application is evident. The effect of CM on functional recovery for a more severe peripheral nerve injury, such as a complete nerve transection, may be even stronger than after the application of GA. This should be taken into account in future studies.

Thus, CM appears to meet the challenge of protecting the nerve from an overwhelming inflammatory response during Wallerian degeneration, on the one hand and preventing further foreign body reactions while the material biodegrades, on the other.

However, we must clearly distinguish between the effects of the material itself and the spacer effect in general. Several approaches, including applying hyaluronic acid after nerve transection [5] or wrapping an autologous vein around the nerve [3], also showed beneficial effects on peripheral nerve regeneration with a minor development of scar tissue formation. Exemplary, Ozgenel et al. wrapped the injured segment of the sciatic nerve with a human amniotic membrane and injected hyaluronic acid into the nerve’s perineural environment, preventing extensive peripheral nerve scarring and adhesion [5].

A possible explanation for this effect was given by Rivlin et al. by estimating a decreased infiltration of fibroblasts and inflammatory cells due to the mechanical barrier [20]. Unfortunately, a direct comparison of the literature’s results to our study is challenging due to the different models to induce peripheral scarring, different evaluation periods, and various assessment methods to evaluate peripheral scarring. Our MRI findings showed that scar tissue formation development is mainly present in the first six weeks (see Fig 3A–3F). Therefore, the ideal spacer must have time-dependent biodegradation that emphasizes its persistence immediately after peripheral nerve injuries in the early phase and its biodegradation in the long term to prevent a foreign body reaction. In addition, the ideal spacer should construct a barrier that modifies the inflammatory response following peripheral nerve injury and protects the lesion site against extensive scar tissue formation. This study has tested an acellular collagen-elastin matrix that appears to meet these requirements based on its promising histological and functional regeneration. With Matriderm® already being in clinical use as a dermal scaffold, its benefits regarding biocompatibility, degradation, and biomechanical aspects have been demonstrated in clinical studies [21–23]. The extensive experience of CM in clinical use, combined with the translational features of this study serve as a foundation for further studies. On one hand, further studies should examine the potential clinical application of CM in nerve surgery, and on the other hand, the exact molecular and genetic mechanisms of the scar preventive effect are yet to be studied by further molecular or in-vitro studies.

Most studies focus on preventing extensive scar tissue formation and the resulting nerve compression. However, in clinical practice, direct nerve adhesion to its surrounding tissue can cause severe pain and discomfort for the patient due to the loss of the gliding potential of the nerve [24]. It was already shown that the additional application of nerve conduits could also positively affect the adhesion of the peripheral nerve. Shintani et al. produced perineural scarring by coagulation of the neural bed and demonstrated a significantly improved adhesion score in rats treated with a supplemental biodegradable nerve conduit six weeks postoperatively [25]. This is in line with our findings by showing the directed adhesion between the peripheral nerve and the surrounding scar tissue formation in the MR neurography for GA-CM and, in contrast, space between the peripheral nerve and the environment for GA+CM after 6 and 12 weeks (see Figs 3 and 4). Due to the considerable decrease in the ratio of connective tissue between GA+CM and GA-CM (see Fig 7A), we can also assume a reduction in the nerve’s adhesion to its perineural environment. Furthermore, the absence of strongly significant discrepancies between the muscle weight ratios of the gastrocnemius muscle and the anterior tibial muscle may suggest that functional impairment is not limited by the motor unit but also may be related to a pain-induced adhesion of the peripheral nerve (see Fig 7B).

However, focusing on the functional recovery and the beneficial effects of a supplementary GA+CM application, the impact of nerve adhesion or nerve compression on the functional impairment requires further investigations and clinical evaluation.

Future research should concentrate on the precise adjustment of the role of Wallerian degeneration under the effect of collagen as a material and the spacer technique in general. Different spacer materials have been described in preclinical studies to reduce scarring around peripheral nerves, including autologous transplants [3, 26], and different natural [27, 28] or synthetic materials [25, 29]. However, the transition to the actual clinical use of these materials is limited. Therefore, testing a material that is already well known in clinical use may facilitate this transition. Collagen has been described in previous studies to improve nerve regeneration and reduce scarring around peripheral nerves and generally has good biocompatibility within humans [4, 14–16, 30]. It has furthermore been researched as a wrapping material for tendons and described as preventing adhesions in that use. The described effects around tendons furthermore support the results of this study demonstrating general adhesion preventive effects of collagen wrapping [31, 32].

Nevertheless, scar tissue formation may not only be influenced by the implementation of the spacer material but also on the cellular level. After blocking calcium channels with a calcium antagonist or using TGF-ß antibodies significantly less scar tissue formation after the sciatic nerve injury was found [20]. Developing a bioactive spacer by combining a spacer material with antibodies or medications known to inhibit scar tissue formation may be a future strategy to prevent peripheral scarring [33]. Li et al. described scar preventive effects after separately using a chitosan-based nerve tube and a hyaluronic acid gel, but the best outcomes after combining both methods [27]. This might also be an approach applicable to collagen-based materials like this study’s CM. Thus, the refinement of different spacer materials as well as comparative studies using a number of different materials could be the next step in finding optimal methods for scar prevention.

A limitation of this study is the lack of precise differentiation between the impact of the collagen matrix itself and the spacer implantation technique in general. Therefore, further studies should compare the collagen matrix to other spacer materials in order to further refine the technique and optimize the choice of the used material.

Even though adding a spacer material leads to beneficial effects in peripheral nerve regeneration, the exact potential of the CM compared to other autologous or synthetic materials should be explored in the future. Due to the different study designs, the potential and effectiveness of collagen are hard to define. In addition, the study is constrained by a limited evaluation period of 12 weeks and focuses on acute peripheral nerve trauma in a translational setting. In the future, it will be necessary to consider the long-term effects of supplementary implantation of CM because of the potential to prevent chronic nerve compression and avoid nerve adhesion to the surrounding tissue.

## Conclusion

Overall, we demonstrated that the additional application of CM has beneficial effects not only on the histological level and the quality of peripheral nerve regeneration but also on a quicker functional recovery after peripheral nerve trauma. Due to its straightforward application and promising outcomes, CM may be a valuable material in supporting postoperative peripheral nerve regeneration in a translational context.

## Supporting information

S1 File (PDF)

## Abbreviations

CM: acellular collagen-elastin matrix
GA: Glutaraldehyde
GA + CM: Glutaraldehyde application with supplementary CM
GA–CM: Glutaraldehyde application without supplementary CM
SSI: Static Sciatic Index
TGF-ß: Tumour growth factor ß
