# Supplementary material for: Avoiding scar tissue formation of peripheral nerves with the help of an acellular collagen matrix
Source: PLoS One. 2023 Aug 4;18(8):e0289677. doi: 10.1371/journal.pone.0289677 (PMC10403074; doi:10.1371/journal.pone.0289677)
Supplement: S1 File — (PDF) [file pone.0289677.s001.pdf]

Animal No	1	2	3	4	5	6	7	8	9	10
Group	GA+CM	GA+CM	GA+CM	GA+CM	GA+CM	GA+CM	GA+CM	GA+CM	GA+CM	GA+CM
Con Tis Rat	1,30314573	1,31033488	1,36381229	1,30924429	1,45908397	1,31114079	1,38521498	1,34802312	1,28778025	1,39589598
MW Gast op	1,33	1,436	1,375	1,362	1,444	1,33	1,457	1,32	1,383	1,297
MW Gast cl	1,398	1,402	1,345	1,38	1,42	1,406	1,42	1,305	1,368	1,285
Ratio Gast	0,95135908	1,02425107	1,02230483	0,98695652	1,01690141	0,94594595	1,02605634	1,01149425	1,01096491	1,00933852
MW Tib ant op	0,45	0,473	0,428	0,439	0,421	0,408	0,45	0,433	0,43	0,388
MW Tib ant cl	0,49	0,446	0,423	0,427	0,416	0,425	0,47	0,427	0,425	0,401
Ratio Tib ant	0,91836735	1,06053812	1,01182033	1,02810304	1,01201923	0,96	0,95744681	1,01405152	1,01176471	0,96758105
Fiber dens	10487,06	10978,17	10532,07	11088,31	10703,09	13360,52	11172,65	15465,05	11215,19	13822,14
Axon thick	4,3116	4,0192	4,4934	3,7852	4,1268	4,0830	4,4281	4,4407	4,7539	4,3104
Myel thick	1,7298	1,3191	1,3811	1,1749	1,7727	1,2589	1,4155	1,2972	1,8157	1,7434
Myel fib thick	7,7712	6,6574	7,2556	6,1351	7,6722	6,6009	7,2590	7,0351	8,3830	7,7971
g Ratio	0,5503	0,5997	0,6129	0,6095	0,5339	0,6133	0,6030	0,6241	0,5603	0,5474
VSSI W1	-20,443	-18,393	-26,993	-15,762	-27,130	-15,313	-21,674	-27,477	-18,238	-22,564
VSSI W2	-23,584	-19,806	-15,698	-20,975	-11,969	-19,336	-18,615	-12,926	-17,429	-16,251
VSSI W3	-23,873	-24,092	-17,626	-14,058	-16,973	-14,540	-17,329	-15,706	-16,712	-16,914
VSSI W4	-20,266	-19,436	-13,702	-14,774	-23,129	-17,416	-21,774	-18,546	-16,803	-16,385
VSSI W5	-14,445	-13,112	-11,419	-11,940	-9,289	-15,478	-12,663	-6,258	-10,407	-10,052
VSSI W6	-0,714	-12,039	-22,532	-11,122	-2,704	-6,235	-8,554	-5,782	-6,606	-9,772
VSSI W7	-12,356	-7,178	-11,913	-8,340	-4,082	-7,017	-6,284	-6,001	-6,293	-6,251
VSSI W8	-10,633	-8,808	-9,686	-6,457	-6,328	-2,824	-4,624	-6,643	-5,649	-6,815
VSSI W9	-5,219	-7,838	-7,722	-5,683	-4,727	-4,002	-5,314	-7,624	-6,667	-5,681
VSSI W10	-3,995	-6,284	-7,949	-6,571	-4,574	-2,463	-7,338	-4,294	-3,983	-6,898
VSSI W11	-5,857	-6,225	-5,045	-7,102	-5,598	-4,611	-3,375	-5,494	-4,244	-6,963
VSSI W12	-6,161	-4,558	-4,728	-3,869	-7,386	-4,982	-7,134	-4,729	-5,686	-5,053

Animal No	11	12	13	14	15	16	17	18	19	20
Group	GA	GA	GA	GA	GA	GA	GA	GA	GA	GA
Con Tis Rat	1,50770807	1,46785528	1,59947861	1,37832832	1,42111089	1,27950429	1,82081834	1,48576927	1,81373063	1,40564123
MW Gast op	1,239	1,25	1,35	1,34	1,308	1,33	1,361	1,281	1,274	1,428
MW Gast cl	1,3	1,295	1,43	1,35	1,341	1,36	1,415	1,305	1,325	1,454
Ratio Gast	0,95307692	0,96525097	0,94405594	0,99259259	0,9753915	0,97794118	0,96183746	0,9816092	0,96150943	0,98211829
MW Tib ant op	0,416	0,38	0,42	0,411	0,407	0,403	0,428	0,391	0,402	0,411
MW Tib ant cl	0,432	0,41	0,426	0,424	0,432	0,413	0,434	0,397	0,44	0,456
Ratio Tib ant	0,96296296	0,92682927	0,98591549	0,96933962	0,94212963	0,97578692	0,98617512	0,98488665	0,91363636	0,90131579
Fiber dens	9951,23	8535,36	9106,39	10444,33	8718,98	9619,61	11389,15	12960,82	8065,81	10761,96
Axon thick	3,1759	3,4503	3,5748	3,5051	4,3057	3,6163	3,5427	3,6852	4,0475	3,5155
Myel thick	1,4700	1,6194	1,5165	1,3390	1,1938	1,3350	1,2897	1,2187	1,2626	1,2280
Myel fib thick	6,1159	6,6892	6,6078	6,1831	6,6933	6,2863	6,1221	6,1226	6,5728	5,9716
g Ratio	0,5147	0,5059	0,5379	0,5640	0,6325	0,5677	0,5758	0,5995	0,6052	0,5871
VSSI W1	-20,795	-27,978	-23,182	-30,681	-25,107	-8,022	-18,962	-15,536	-19,724	-13,061
VSSI W2	-24,031	-22,696	-26,693	-22,908	-18,541	-19,295	-17,488	-21,682	-22,451	-20,713
VSSI W3	-40,640	-36,319	-25,778	-26,247	-40,145	-25,160	-33,809	-33,652	-31,994	-28,540
VSSI W4	-33,790	-29,636	-30,527	-33,832	-34,029	-30,811	-30,951	-30,075	-35,492	-27,579
VSSI W5	-26,978	-25,176	-29,487	-26,044	-25,069	-21,974	-29,204	-24,315	-25,686	-25,791
VSSI W6	-27,419	-26,346	-24,883	-27,349	-25,700	-23,635	-22,307	-22,769	-23,873	-35,404
VSSI W7	-21,877	-23,565	-18,626	-17,666	-17,215	-16,222	-15,875	-18,002	-15,480	-24,355
VSSI W8	-14,049	-14,401	-14,461	-13,485	-15,517	-12,904	-11,209	-14,360	-12,858	-17,352
VSSI W9	-12,102	-9,589	-7,180	-10,444	-10,452	-9,018	-7,423	-10,722	-9,180	-8,814
VSSI W10	-10,268	-8,986	-6,651	-7,408	-6,560	-8,567	-6,936	-6,508	-5,007	-6,170
VSSI W11	-6,635	-6,706	-5,435	-6,272	-5,165	-6,421	-5,835	-4,354	-5,288	-5,410
VSSI W12	-5,089	-5,723	-4,952	-6,130	-6,005	-4,063	-6,505	-3,963	-4,922	-6,140

Animal No	21	22	23	24	25	26	27	28	29	30
Group	Sham	Sham	Sham	Sham	Sham	Sham	Sham	Sham	Sham	Sham
Con Tis Rat	1,33171999	1,36679082	1,20056933	1,26691251	1,27671845	1,19157570	1,47671433	1,33506355	1,27527914	1,23143404
MW Gast op	1,437	1,475	1,446	1,359	1,293	1,37	1,338	1,303	1,357	1,32
MW Gast cl	1,423	1,462	1,153	1,357	1,3	1,368	1,336	1,309	1,378	1,312
Ratio Gast	1,00983837	1,00889193	1,25411969	1,00147384	0,99461538	1,00146199	1,00149701	0,99541635	0,98476052	1,00609756
MW Tib ant op	0,422	0,458	0,48	0,404	0,41	0,447	0,425	0,441	0,423	0,397
MW Tib ant cl	2,86	2,937	2,599	2,716	2,593	2,738	2,674	2,612	2,735	2,632
Ratio Tib ant	0,14755245	0,15594144	0,18468642	0,14874816	0,15811801	0,16325785	0,15893792	0,16883614	0,15466179	0,15083587
Fiber dens	11005,51	10774,87	10908,17	14883,45	12519,97	11070,00	12452,73	11324,95	10371,63	11280,96
Axon thick	4,6270	4,1943	4,2900	3,3632	4,6534	4,7199	4,2937	3,9215	4,2174	4,2243
Myel thick	1,5743	1,4677	1,5000	1,2161	1,6025	1,6075	1,5063	1,3662	1,5317	1,4573
Myel fib thick	7,7757	7,1296	7,2900	5,7954	7,8585	7,9350	7,3062	6,6539	7,2808	7,1389
g Ratio	0,5919	0,5821	0,5800	0,5746	0,5830	0,5864	0,5806	0,5845	0,5717	0,5861
VSSI W1	-8,905	-5,784	-5,767	-6,612	-4,029	-7,077	-5,064	-5,226	-6,827	-1,944
VSSI W2	-6,231	-5,642	-7,921	-3,723	-7,785	-7,959	-3,096	-5,591	-7,370	-6,575
VSSI W3	-5,494	-7,941	-6,628	-5,160	-5,205	-6,878	-3,940	-3,904	-5,574	-4,148
VSSI W4	-4,569	-8,186	-3,178	-7,566	-5,975	-6,462	-4,252	-3,542	-7,879	-4,017
VSSI W5	-5,374	-6,146	-3,697	-6,084	-6,943	-7,804	-7,855	-5,840	-8,359	-3,307
VSSI W6	-6,311	-7,315	-6,891	-2,962	-8,358	-5,224	-7,332	-4,433	-6,624	-3,333
VSSI W7	-7,473	-5,651	-5,054	-7,128	-3,404	-6,460	-4,279	-4,012	-3,439	-3,285
VSSI W8	-6,875	-6,118	-6,983	-6,572	-2,411	-4,948	-1,149	-4,710	-7,778	-6,595
VSSI W9	-6,606	-6,557	-5,857	-3,478	-3,823	-6,277	-4,137	-5,723	-6,469	-3,122
VSSI W10	-6,085	-6,403	-4,902	-4,408	-4,723	-5,884	-2,383	-5,433	-6,213	-3,778
VSSI W11	-6,624	-4,748	-4,398	-4,064	-4,198	-6,786	-4,335	-5,587	-4,591	-6,100
VSSI W12	-6,148	-4,827	-2,940	-4,168	-5,171	-6,387	-3,320	-6,197	-4,471	-5,499
